# The Role of MicroRNAs in Recurrence and Metastasis of Head and Neck Squamous Cell Carcinoma

**DOI:** 10.3390/cancers11030395

**Published:** 2019-03-21

**Authors:** Chris X. Yang, Wafik Sedhom, John Song, Shi-Long Lu

**Affiliations:** 1 Department of Otolaryngology, University of Colorado Anschutz Medical Campus, Aurora, CO 80045, USA; chris.yang1996@yahoo.com (C.X.Y.); wafik.sedhom@ucdenver.edu (W.S.); john.song@ucdenver.edu (J.S.); 2 Department of Dermatology, University of Colorado Anschutz Medical Campus, Aurora, CO 80045, USA; 3 Department of Pathology, University of Colorado Anschutz Medical Campus, Aurora, CO 80045, USA

**Keywords:** HNSCC, microRNA, metastasis, EMT, CSC

## Abstract

Head and neck squamous cell carcinoma (HNSCC) affects 650,000 people worldwide and has a dismal 50% 5-year survival rate. Recurrence and metastasis are believed the two most important factors causing this high mortality. Understanding the biological process and the underlying mechanisms of recurrence and metastasis is critical to develop novel and effective treatment, which is expected to improve patients’ survival of HNSCC. MicroRNAs are small, non-coding nucleotides that regulate gene expression at the transcriptional and post-transcriptional level. Oncogenic and tumor-suppressive microRNAs have shown to regulate nearly every step of recurrence and metastasis, ranging from migration and invasion, epithelial-mesenchymal transition (EMT), anoikis, to gain of cancer stem cell property. This review encompasses an overview of microRNAs involved in these processes. The recent advances of utilizing microRNA as biomarkers and targets for treatment, particularly on controlling recurrence and metastasis are also reviewed.

## 1. Introduction

Head and neck squamous cell carcinomas (HNSCCs) arise in the squamous epithelium along the head and neck region, including the nasal cavity, oral cavity and tongue, pharynx (nasal pharynx, oropharynx, hypopharynx) and larynx. They affect 650,000 people and claim 350,000 lives worldwide annually [1]. In the United States alone, there are some 55,000 new cases of HNSCC and 15,000 deaths every year [2]. Tobacco, alcohol, and human papilloma virus (HPV) infection are known risk factors for HNSCC [3,4]. Tobacco and alcohol contribute to around 75% of HNSCC cases, and HPV infection to at least 25% of cases. [5,6] HNSCC is still a deadly cancer, with an average 50% overall 5-year survival rate [7]. The main reasons for the poor prognosis of HNSCC patients are loco-regional invasions, treatment-resistance, recurrence, and metastasis [8]. On initial presentation, ~10% of HNSCC cases already show metastases and the survival rate for these patients is less than one year [9]. Additionally, ~30–40% of post-treated HNSCC patients develop recurrence or metastasis [9]. Thus, therapies controlling HNSCC recurrence and/or metastasis are pivotal to improve poor survival of HNSCC patients. 

MicroRNAs (miRNA) are small, non-coding molecules of about 19–25 nucleotides in length that regulate gene expression at the transcriptional and post-transcriptional level [10]. Originally discovered in *Caenorhabditis elegans* over twenty years ago, they have since been linked with regulating 60% of the genes in the human genome [11]. The miRNA gene is initially transcribed within the nucleus. After processing and cleavage, the pre-miRNA is exported to the cytoplasm, where it matures. They are then incorporated within an RNA-induced silencing complex and induce post-transcriptional gene silencing by base pairing with a target mRNA and interfering with its translation to protein [12]. During this process, the miRNA may promote the degradation of the mRNA or disassembly of the ribosome complex. This particular function of miRNAs enables them to regulate many cellular pathways, especially those involved with cancer. The genes that miRNAs regulate get involved in all most all cancer hallmarks related to recurrence and metastasis, including, but not limiting to proliferation, apoptosis or anoikis, epithelial-mesenchymal transition (EMT), and cancer stem cells (CSCs) [13].

Metastasis is complex, and rate-limiting process, including tumor cells disseminate initially from primary site, invade into blood vessels (intravasation), survive in blood stream (resistance to a special form of apoptosis, i.e., anoikis), invade out of blood vessel (extravasation), and survive and colonize in distant sites. miRNAs are shown to be involved nearly in all the steps of metastasis. The following review will focus on: (1) miRNAs in several steps critical for metastasis and recurrence, i.e., EMT, migration and invasion, anoikis and CSC; (2) grouping miRNAs as promoter or suppressor based on their functional role in metastasis and recurrence and (3) summarizing the reported miRNAs either as biomarkers for diagnosis or as target for treatments to control HNSCC recurrence and metastasis. 

## 2. miRNAs Involved in Pathological Processes of Metastasis and Recurrence

### 2.1. Migration and Invasion 

Migration and invasion is the initial step in the metastatic process, with the cardinal event involving EMT (discussed in the next section) that allows the cells to become more motile by becoming mesenchymal cells. Here is a summary of examples of migration and invasion that are independent of EMT. Koshizuka et al. found that due to miRNA-199’s absence in HNSCC, the gene ITGA3 is allowed to proliferate and cause dysregulation of the extracellular matrix (ECM) and contributes to oncogenic signaling. When this gene was knocked down, migration and invasion were reduced [14]. miRNA-138 has been shown to act upon RhoC to exert its function in migration and invasion. MiRNA-138 has previously been associated with cell migration, EMT, and cell cycle dysregulation. They have shown that RhoC regulates stem-cell transcription factors via signal transducer and activator of transcription 3 (STAT3) in the IL-6 signaling pathway [15]. The miRNA-29 family has been consistently downregulated in HNSCC. In one study performed by Kinoshita et al., they determined that miRNA-29s primarily interacts with laminin-332 and *α*6*β*4 integrin, ECM components that are important for proper adhesion of cells [16]. MiRNA-329 and miRNA-410 have been shown to have an inverse correlation with Wnt-7b, a protein within the Wnt/β-catenin pathway. They are implicated in oral SCC (OSCC) pathogenesis due to their interactions with Meg-3, which is low in cancer cells and leads to upregulation of Wnt signaling [17]. 

The Chang et al. group identified miRNA-376c-3p as a tumor suppressor miRNA. When it is lost, RUNX2 expression increases which in turn activates INHBA. This increases the invasive and migratory abilities as well as infiltration to lymph nodes [18]. Addition of this miRNA reduced these metastatic effects. Geng et al. were the first to associate the miRNA-365a-3p with laryngeal squamous cell carcinoma (LSCC). They determined that its overexpression upregulates p-AKT (Ser473) to promote metastasis [19]. Fukumoto’s group identified miRNA-26a/b, miRNA-29a/b/c, and miRNA-218 as direct inhibitors of Lysyl oxidase like 2 (LOXL2). Although function is still not fully understood, it is thought that the lysyl oxidase (LOX) family of proteins is responsible for ECM remodeling in conjunction with matrix metalloproteases (MMP) in a way that promotes cancer metastasis [20]. In addition, LOXL2 has been shown to interact with Snail1, one of the transcription factors involved in EMT, and thus may play an active role in the cells being able to undergo this important cancerous process [21].

### 2.2. EMT and CSC

EMT is a critical process for metastasis or recurrence of cancer by conferring cell abilities ranging from enhancing migration and invasion, resistance to anoikis, and CSC properties to regenerate new tumors either at the same site (recurrence) or distant site (metastasis). EMT also confers resistance of chemo- or radiation therapies. 

miRNA-34a is significantly reduced in HNSCC and known transcription factors of EMT and CSCs such as Nanog, Sox2, Oct3/4, and Aldehyde dehydrogenase (ALDH) were upregulated. When miRNA-34a mimics were placed into the CSCs, a decrease in the sphere-forming capacity and transcription factors were detected. These interactions may be mediated by interactions with proteins Snail and Twist. This supports the role of miRNA-34a as a potential tumor-suppressor in HNSCC. In addition, they observed a correlation between HPV+ HNSCC and higher ALDH1A1 levels, suggesting that HPV+ tumors portend a worse prognosis and higher activity of CSCs [22]. Obayashi et al. observed a correlation between the presence of miRNA-203 (in cooperation with other members of the miRNA-200 family) and the delay of EMT progression as monitored by downregulation of E-cadherin and upregulation of N-cadherin. This has been postulated in other cancers to work by suppression of transcription factors SNAI1, SNAI2, ZEB2, and VEGFA [23]. In addition, they isolated gene product NUAK1 as directly upregulated by the downregulation of miRNA-203, suggesting that NUAK1 has EMT-promoting properties. The downregulation of miRNA-203 was observed in HNSCC as a result of hypermethylation [24]. Liu et al. analyzed the role of miRNA-138 in HNSCC since it has been downregulated in many cancer types. The expression of EMT transcription factor E-cadherin was reduced while vimentin expression increased, which supports the formation of mesenchymal cells. They further found that miRNA-138 allows for these effects through interactions with the genes Vimentin (VIM), ZEB2, SNAI2 and EZH2 in three distinct pathways [25]. Tan et al. observed the gene (Metadherin) MTDH is allowed to proliferate when miRNA-98 is downregulated, as it is in HNSCC. This in turn leads to EMT. Although not fully elucidated, it is believed that MTDH exerts its effects by modulating the PI3K/AKT pathway, one of the most commonly affected pathways in HNSCC [26]. Snail and Slug are other key mediators of EMT. Zheng et al. found that these two proteins overexpress EZH2 via downregulation of miRNA-101 in OSCC. In this context, EZH2 has an oncogenic role, countering some evidence in other malignancies that it is a tumor suppressor [27].

Other experiments have attempted to look at the use of drugs on miRNA expression. Liu et al. tested the drug sophocarpine in HNSCC and determined that miRNA-21 is significantly downregulated when the drug is introduced to these cancer cells. Transfection with miRNA-21 mimics along with sophocarpine resulted in decreased cell viability, invasion, and migration via inhibition of Dicer processing, affecting the downstream products vimentin and E-cadherin in opposite ways [28]. Sun et al. were the first to link miRNA-21, a known causative miRNA of multiple malignancies, to the overactivation of cyclin-dependent kinase 5 through the STAT3/miRNA-21 pathway, which was shown to promote EMT. In addition, reversal of EMT was seen with a STAT3 inhibitor [29].

Interest is growing in maximizing combinations of therapies for HNSCC treatment, including radiotherapy. De Jong et al. focused on the biologic basis of radioresistance in tumors based on the ability of miRNAs to promote/inhibit EMT as a prognosticator of treatment efficacy. They identified several miRNAs that, due to downregulation in HNSCC, could not regulate mRNAs involved in EMT promotion. Of those identified, miRNA-203 seemed to be the most influential. Thus, radioresistant laryngeal carcinomas tended to be tumors in which these miRNAs were lost [30].

### 2.3. Anoikis 

Anoikis is the process by which an anchorage-dependent cell, when detached from the ECM, undergoes a programmed cell death in order to minimize the aberrant cells that proliferate outside their environment. Although direct links between miRNA and anoikis properties still have yet to be elucidated, some extrapolations can be made. Several proteins, including Phosphatase and tensin homolog (PTEN), AKT, and B cell lymphoma 2 (Bcl-2), have been linked to anoikis. PTEN, a molecule that acts as an inhibitor to the PI3K pathway, is affected by miRNA-21, miRNA-214, miRNA-744-3p, and miRNA-205. By reducing PTEN expression, it allows for upregulation of central components to the PI3K pathway such as AKT, and this unchecked proliferation inhibits anoikis [26]. An additional array of four miRNAs in OSCC (miRNA-96-5p, miRNA-21-3p, miRNA-141-3p, and miRNA-130b-3p) were also implicated in decreased PTEN expression [31]. Bcl-2, an anti-tumor mitochondrial factor, is also implicated in anoikis. Its overexpression in cancer has been associated with anoikis resistance. miRNA-371-5p is seen as a potential stimulant of anoikis since higher levels of the miRNA correlated with lower levels of Bcl-2. [32] Survivin, an anti-apoptotic protein identified in several cancers to inhibit anoikis, may also be regulated by miRNA-34a. It has been hypothesized that miRNA-34a may downregulate cell proliferation in HNSCC through the inhibition of survivin [33]. Indeed, survivin expression in HNSCC is associated with metastasis and poor prognosis, and several studies showed that miRNA-34a overexpression markedly downregulated survivin [34,35].

### 2.4. HNSCC Recurrence 

In SCC of the oropharynx (OPSCC), a study by Zhu et al. looked at SNP in miRNA-binding sites in the 3′ UTRs of genes that may indicate the likelihood of recurrence. *BRCA1* and *RAD51* are genes that are required for double strand break (DSB) repair of DNA. In this study, it was determined that variants of *BRCA1* (rs12516) and *RAD51* (rs7180135) had better disease-free survival and lower recurrence risk than those patients with the normal variant of the gene because these genes have roles in repairing the DSB that aid in creating malicious cancer cells. The miRNA binding regions thus prove important in determining the expression of these genes and the outcome of cancer recurrence [36].

## 3. MicroRNAs as either Promoters or Suppressors of Metastasis.

MicroRNAs are categorized as either oncogenic or tumor suppressors based on their role in metastasis. In Table 1 and Table 2, we summarize some of the miRNAs that upregulate and downregulate, respectively, HNSCC metastasis. Of particular interest are the mechanisms by which these miRNAs regulate metastasis.

### 3.1. MiRNAs that Promote HNSCC Metastasis 

Table 1 shows the miRNAs that upregulate metastasis in HNSCC. However, they exert their effects through different targets and pathways. This provides an in-depth look at the mechanisms by which they function, and also provides an expedited way to select the relevant miRNA(s) for combined treatment in future.

As an important miRNA in promoting the proliferation of lung cancer [49], miRNA-134 has also been shown to be highly expressed in HNSCC patients with lymph node metastasis (LNM) than those without. In mice models, miRNA-134 expressing tumors had significantly higher neck nodal metastasis relative to control tumors. In fact, miRNA-134 expression enhanced primary tumorigenesis along with metastasis of HNSCC. The target of miRNA-134 was identified as WW domain-containing oxidoreductase (WWOX), a tumor suppressor that is part of the family of WW domain-containing proteins. Transforming growth factor-β (TGF-β) controls cell growth and death through signaling with WWOX, and deficiency in this signaling facilitates cancer growth [50]. In this case, miRNA-134 expression enhances metastasis of HNSCC by downregulating WWOX mRNA. This is further confirmed when ectopic WWOX expression was shown to decrease the invasion of HNSCC cells with miRNA-134 expression [37].

In patients with OSCC, high miRNA-155-5p expression was positively correlated with LNM. In culture, the migratory ability of human OSCC cells were increased by miRNA-155-5p mimic and decreased when transfected with mR-155-5p inhibitor. Also, when transfected with miRNA-155-5p inhibitor, E-cadherin, an epithelial biomarker, was upregulated. However, N-cadherin and vimentin, both mesenchymal biomarkers, were downregulated [51]. This is indicative of cells undergoing EMT, and it is later implied that miRNA-155-5p may induce EMT by upregulating STAT3 via SOCS1 downregulation [38].

Another oncogenic miRNA of interest is miRNA-196b, which has been dysregulated in a variety of cancers, including that of the colon, esophageal, lung, and pancreas. During tumorigenesis, miRNA-196b promotes tumor proliferation and invasion. [52,53,54,55] In LSCC patients, miRNA-196b is upregulated compared to control specimens, while SOCS2 expression was decreased 52%. By knocking down miRNA-196b, migration and invasion was observed to decrease. SOCS2 overexpression was also associated with decreased invasion and migration. By binding to the 3′UTR of SOCS2, miRNA-196b inhibits SOCS2. This promotes LSCC cell proliferation and invasion, and suppresses apoptosis. [39] Other studies show that epigenetic regulation of miRNA-196b expression by more frequent hypomethylation of CpG islands upstream of the miRNA-196b gene promoted OSCC migration and invasion abilities [56].

Microarray profiling showed that miRNA-744-3p was significantly upregulated in LSCC tissue with cervical LNM. Mice injected with miRNA-744-3p suppressed LSCC cells exhibited fewer metastatic cancer nodules when compared with the control group. To exert its effects, miRNA-744-3p targets PTEN and PDCD4. Previous studies have shown that PTEN could suppress migration and invasion in LSCC [57]. PDCD4 upregulation could inhibit the invasive behaviors of oral cancer [58]. Further downstream of PDCD4 and PTEN is MMP-9, which is upregulated in LSCC tissues, associated with LNM of LSCC patients, and develops the EMT phenotype in LSCC [59]. PDCD4 is capable of suppressing MMP-9 expression. As such, following miRNA-744-3p inhibition of PDCD4 and PTEN expression, MMP-9 is enhanced, leading to EMT and migration of LSCC [40].

### 3.2. MiRNAs that Suppress HNSCC Metastasis

Tissue samples from LSCC patients with distant metastases revealed that miRNA-145-5p expression was significantly lower, caused by hypermethylation of the miRNA-145-5p proximal promoter. The target of interest is fascin actin-bundling protein 1 (FSCN1), an actin-filament bundling oncogene that has been studied in various cancers [60]. FSCN1 is frequently upregulated in LSCC tissues, and when its expression levels are high, it is often contrasted by low miRNA-145-5p expression levels. By using mice models, it was determined that miRNA-145-5p directly targets the 3′UTR region of FSCN1 mRNA. By suppressing FSCN1, miRNA-145-5p inhibited cell migration and invasion in Transwell assays. In fact, FSCN1 knockdown also inhibited cell proliferation and increased cleavage of caspase-3. LSCC cells transfected with miRNA-145-5p mimic and FSCN1 siRNA displayed increased expression of epithelial markers and decreased expression of mesenchymal markers, indicating that miRNA-145-5p inhibits EMT, while FSCN1 promotes EMT [42].

Integrins regulate the attachment of cells to the extracellular matrix. Dysregulation of integrin expression may contribute to cancerous states, as is the case with integrin α3 (ITGA3) [61], whose overexpression was confirmed in HNSCC specimens. ITGA3 is directly regulated by all members of the miRNA-199 family, which includes miRNA-199a-5p, miRNA-199a-3p, miRNA-199b-5p, and miRNA-199b-3p. In HNSCC cell lines, the miRNA-199 family exhibits significantly lower expression levels. Transfection of any member of the miRNA-199 family into HNSCC cell lines inhibited cell migration activity. Restoration of miRNA-199 represses ITGA3 mRNA expression, which in turn inhibited HNSCC cell migration and invasion. [14]

In different regions of HNSCC, certain miRNAs can also have distinct targets. For instance, miRNA-203 functions differently in hypopharyngeal cancer (HPC) and OSCC. Previously, miRNA-203 has been shown to suppress cell proliferation in lung and esophageal cancer [62,63]. In human specimens of HPC, miRNA-203 expression levels were lower than those of normal specimens. Additionally, low levels of miRNA-203 was associated with LNM. miRNA-203 targets podoplanin (PDPN), a protein that promotes tumor cell-induced platelet aggregation, which enhances tumor metastasis [64]. By overexpressing miRNA-203 in HPC cells through transfection and downregulating PDPN, it was determined through wound-healing and invasion assays that these cells had slower cell migration and less cell invasion than control cells [64]. Alternatively, in OSCC, miRNA-203 targets NUAK1, a molecule associated with the invasion of breast and lung cancers [65,66]. When NUAK1 is transfected into OSCC cells, tumor invasion increased. Cells with miRNA-203 downregulation and NUAK1 overexpression resulted in EMT. Restoration of miRNA-203 suppressed NUAK1, which decreased tumor invasion and LNM [66]. 

Within HNSCC, certain miRNAs may also target the same molecule. For instance, miRNA-363 is downregulated in HNSCC with LNM. Similar to miRNA-203, mentioned above, miRNA-363 also targets PDPN. In fact, the 3′UTR of the PDPN gene has three binding sites for miRNA-363. Inhibition of PDPN by miRNA-363 suppresses HNSCC migration and invasion [47].

## 4. miRNAs as Biomarkers for HNSCC

### 4.1. Diagnostic Biomarker 

Most biomarker studies are still limited to the research phase and there are no biomarker-based assays for early detection in HNSCC patients in clinic. One common mechanism responsible for reduced or loss of miRNA expression is epigenetic silencing of miRNA genes by DNA methylation, a process that occurs early in tumorigenesis [67]. Across various human cancers, more than 20 miRNAs have been reported to be silenced by DNA methylation [68,69]. As such, DNA methylation serves as a diagnostic biomarker for human cancers, and such processes for the detection of HNSCC have been developed [70].

For instance, miRNA-9 had previously been shown to serve as a tumor suppressor in HNSCC [71,72,73]. It directly targets the CXC chemokine receptor 4 (CXCR4) gene, a G-protein-coupled receptor that is commonly involved with tumor metastasis [74]. miRNA-9 inhibits the migratory and invasive capabilities of HNSCC cells by suppressing CXCR4. Our lab has identified that the genomic loci encoding miRNA-9 were methylated in a subset of human HNSCC tissue samples, resulting in miRNA-9 silencing [75]. To further identify genomic loci encoding miRNA, which is methylated specifically in HNSCC, we have screened genomic loci for miRNA in HNSCC cell lines and tissue and identify a panel of methylated genomic loci for miRNA (mg-miRNAs) as a biomarker for HNSCC. The robustness of these mgmiRNAs as biomarkers and sensitivity of detection technology made it detectable in patients’ saliva. Clinical utilizes of using this panel of mgmiRNA markers in HNSCC patients’ follow-up of recurrence and metastasis is undergoing in our lab [76].

### 4.2. Treatment and Prognostic Biomarkers

Despite advancements in the efficacy of treatment, the prognosis of advanced HNSCC is poor, commonly jeopardized by treatment resistance. The altered expression of miRNAs has been correlated with resistance to chemotherapy and radiotherapy. Recent studies have demonstrated the potential of miRNAs to serve as biomarkers for predicting the post-therapy outcomes of HNSCC patients. 

Resistance to anticancer chemotherapy is the primary contributing factor to treatment failure, which exacerbates prognosis of cancer patients. This typically results from alterations of different molecular pathways. MiRNAs have been hypothesized to play a role in chemoresistance. When comparing human cisplatin-sensitive tongue squamous cell carcinoma and cisplatin-resistant cell lines, there were significant increased levels of miRNA-23a, miRNA-214, miRNA-518c, miRNA-608, and the let-7 family of miRNAs. It is proposed that miRNA-214 induces cell survival and cisplatin resistance by downregulating the gene responsible for coding the PTEN protein, which leads to activation of the AKT pathway. In addition, there were decreased levels of miRNA-21 and miRNA-342 [77]. miRNAs also play a role in the radioresistance of HNSCCs. For instance, miRNA-125b was found to be downregulated in OSCCs. Transfection of OSCC cells with miRNA-125b enhanced radiosensitivity, likely through suppression of ICAM2, a protein that facilitates survival by activating the PI3K pathway. MiRNA-125b was also shown to be correlated with survival and radiotherapy response, and hence can be used as a prognostic marker in OSCC [78].

Several studies have also employed miRNA expression profiling to determine miRNAs with prognostic potential. In comparing 150 OPSCCs, miRNA-31 and miRNA-24 were associated with poor prognosis. Conversely, miRNA-146a was associated with favorable prognosis [79]. Through analyzing primary HNSCC tissue samples with and without LNM, it was found that expression levels of miRNA-26 were higher in metastatic HNSCC and associated with N stage, poor differentiation, and recurrence. Interestingly, miRNA-125b expression levels were elevated in primary HNSCC, contrary to results from the aforementioned study. MiRNA-125b was also associated with N stage, metastasis, and death in HNSCC patients [80]. These conflicting results demonstrate the variability of miRNA expression in different regions of HNSCC. In OSCC, miRNA-125b may be downregulated, but in HNSCC, miRNA-125b expression is not only higher in the metastatic primary tumor compared with non-metastatic tumors, but also higher in the secondary tumor. Based on the presentation of miRNA-125b in metastatic HNSCC tumors, we speculate that it may serve as a prognostic biomarker for cancer staging.

The Let-7 miRNA family is commonly implicated in HNSCC and has been widely characterized in various studies. Meta-analyses of miRNAs in HNSCC prognosis reveal that decreased expression of miRNA-Let-7d and Let-7g are particularly associated with poor prognosis [81]. Underexpression of Let-7 was also correlated with progression to metastatic HNSCC tumors [82], and one unit decrease in Let-7g expression increased the risk of OSCC recurrence by 2.6-fold and the risk of death by 12.9-fold [83].

### 4.3. HNSCC Recurrence Biomarkers

Hess et al. have refined five miRNAs that, in conjunction, may be helpful in predicting recurrence of HNSCC. They analyzed the miRNAs (let-7g-3p, miRNA-6508-5p, miRNA-210-5p, miRNA-4306, and miRNA-7161-3p) and divided the patients into high and low-risk groups based on their expression of these. The high-risk group had a 70% chance of recurrence while the low-risk group had about a 30% chance [84]. These signatures have future potential as a guide to pursue aggressive vs. less aggressive chemotherapy, radiotherapy, and/or surgery. 

Ganci et al. looked at a combination of four miRNAs (miRNA-21-3p, miRNA-21-5p, miRNA-96-5p, miRNA-429) in the area surrounding the tumor and its presence indicated higher risk of recurrence. Two of these four have been observed in oral leukoplakia, a premalignant lesion. These miRNAs did not correlate with tumor recurrence in otherwise healthy tissue [85]. The same group saw higher expression of four miRNAs (miRNA-96-5p, miRNA-21-3p, miRNA-141-3p, and miRNA-130b-3p) that predicted lower recurrence-free survival. The lab then tested the miRNAs in OSCC Cal27 cells and, by inhibiting them, noticed there was a decrease in both the Cal27 cells and a key cell cycle protein, cyclin D1. They also noticed that by suppressing these miRNAs, there was an upregulation of important proteins such as PTEN and E-cadherin. The loss of these are associated with tumor progression and EMT [31].

Some miRNAs can be specifically used to monitor post-surgical recurrence as well. Yan et al. looked at 20 OSCC patients post-operatively, with a focus of three upregulated miRNAs (miRNA-148a-3p, miRNA-26a-5p and miRNA-21-5p) and three downregulated miRNAs (miRNA-375, miRNA-92b-3p and miRNA-486-5p). The data showed a significant difference between the control group and the post-op group (9-12 months following surgery) for miRNA-486-5p, miRNA-375 and miRNA-92b-3p, with the downregulation of these indicating stronger OSCC recurrence [86].

### 4.4. Metastatic Biomarkers

The utility of miRNAs as a marker for the presence/absence of cancer spread is an exciting, burgeoning field. de Carvalho et al. lab identified miRNA-203 and miRNA-205 which were extremely sensitive markers for the detection of cervical lymph node metastases, even isolated cancer cells. This has the benefit of being able to accurately stage patients with FNA without the tedious, time-consuming process of immunohistochemical staining [87]. Another potential marker is miRNA-491-5p, which was shown to be decreased in OSCC. They usually bind to GIT1, and the elevation of GIT1 correlated with lymph node metastasis and thus tumor grade [88].

A vastly understudied area is the use of miRNAs as biomarkers for HNSCC metastasis. In OSCC, tumors with low levels of miRNA-491-5p were more prone to develop LNM. Overexpression of miRNA-491-5p significantly inhibited migration and invasion of OSCC cells, and mice that were transfected with miRNA-491-5p experienced lung tumor areas that were reduced to 45% of the control [88].

Underexpression of Let-7, miRNA-155, and miRNA-146a are all correlated with progression to metastatic tumors. MiRNA-375, mentioned above as a diagnostic biomarker of HNSCC, is reported to have the most significantly lowered expression levels in HNSCC tumor samples. Additionally, regardless of the site or stage of the tumor, incidence of distant metastasis was correlated with lower expression levels of miRNA-375. By inducing overexpression of miRNA-375 in HNSCC cell lines, invasion in the presence of epidermal growth factor (EGF) was reduced by 40% [89].

Although studies regarding this topic are relatively scarce, many of the miRNAs we have identified in Table 1 and Table 2 may have the potential to serve as metastatic biomarkers. In fact, miRNA-205 and miRNA-203, which was extensively discussed in Table 2, are capable of identifying all metastatic samples of HNSCC, regardless of the size of the metastatic deposit and the time of sample collection [87].

## 5. Treatment

In HNSCC, metastasis and recurrence contribute significantly to the poor survival. Therefore, exploring methods of controlling metastasis and recurrence, has been a major goal in cancer research. Certainly, because of the complex relationship between miRNAs and their target molecules, identifying miRNAs that play a significant role in regulating metastasis remains challenging. Although tumor suppressor miRNA-34 is the only miRNA in clinical trials, progress in identifying miRNA targets that control HNSCC metastasis and development of new treatment techniques have been made. Some miRNAs have been identified to be directly involved with radioresistance and chemoresistance, these miRNAs have the potential to be used as drug targets in combination with radiotherapy and chemotherapy. 

### 5.1. MiRNAs Involved with HNSCC Radioresistance

Ataxia-Telangiectasia Mutated (ATM) kinase is a serine/threonine protein kinase that is activated in the presence of DNA double-strand breaks. ATM-deficiency leads to radiosensitivity, a process that is regulated by proteins such as SMC1. Dysregulation of ATM function leads to upregulation of miRNA-16, miRNA-29b, miRNA-150, and miRNA-1254, while Let-7e is downregulated [90]. An examination of the pathway reveals that SMC1A, part of the SMC1 protein family, is regulated by Let-7e. Bcl-2 and MCL1, both anti-apoptotic factors, are regulated by miRNA-16 and miRNA-29b in leukemia and glioblastoma, respectively. [91,92] In HNSCC patients who respond favorably to radiation therapy, miRNA-16 and miRNA-29b are upregulated.

Another important miRNA, miRNA-196a, serves as an oncogenic miRNA in HNSCC, which is similar to the role of its family member, miRNA-196b, as mentioned in Table 1. Not only are HNSCC cells that overexpress miRNA-196a more radioresistant, metastatic abilities are also enhanced. Patients with recurrent HNSCC after treatment exhibit higher levels of miRNA-196a, with trends analogous to that of OSCC patients [93]. It was determined that endogenous levels of miRNA-196a and Annexin A1 (ANXA1) were inversely correlated. Further, miRNA-196a was found to directly suppress ANXA1. Given that the expression of ANXA1 is correlated with less aggressive tumors, suppression of ANXA1 is associated with tumor progression and metastasis [94]. Unsurprisingly, ANXA1 silencing induces the same effects in the HNSCC cell lines as that of miRNA-196a overexpression. Namely, radiation resistance, cell proliferation, and increased migration [95].

### 5.2. MiRNAs Involved with HNSCC Chemoresistance

Compared to miRNAs involved with radioresistance, fewer miRNAs that regulate chemoresistance have been found. Previously, it was determined that miRNA-125b enhances radiosensitivity in OSCC patients, but serves as a prognostic biomarker in metastatic HNSCC [80,82]. Its behavior in HNSCC appears to be dependent on the region of the head and neck. 

Another member of the miRNA-125 family, miRNA-125a, regulates cisplatin response in LSCC. Expression of miRNA-125a is decreased not only in LSCC tissues, but also in laryngeal CSCs. Laryngeal CSCs were initially determined to be resistant to cisplatin treatment, but overexpressing miRNA-125b enhanced cisplatin-induced cell death in the CSCs. In mice, enforced miRNA-125a expression increased the anti-tumor effect of cisplatin. Specifically, miRNA-125a targets the Hematopoietic cell-specific protein 1-associated protein X-1 (HAX-1), a marker that has been previously correlated with lymph node metastasis in NPC [96]. Overexpression of HAX-1 inhibited the cell death induced by combining cisplatin and miRNA-125a treatments. Moreover, while miRNA-125a and cisplatin cause cytochrome c to be released from the mitochondria of laryngeal CSCs and subsequent activation of caspase-9 and caspase-3, HAX-1 inhibits mitochondria-initiated apoptosis. Inhibition of HAX-1 by miRNA-125a helps to counter multiple drug resistance (MDR) in laryngeal CSCs, with decreased IC50 levels of vincristine, etoposide, and doxorubicin [97].

## 6. Conclusions

HNSCC cells with abnormal miRNA expression evolve different capabilities for recurrence and metastasis through EMT, increase of migration and invasion, aniokis, or gaining of CSC properties. Identification of miRNAs and understanding the role of miRNAs in these process will not only reveal the molecular mechanisms of HNSCC progression, but also yield novel candidates for early detection of HNSCC recurrence and metastasis. Moreover, targeting miRNAs and their downstream signaling pathways or targets is promising for development of novel therapy for HNSCC, particularly on controlling recurrence and metastasis of this devastating disease.

## Figures and Tables

**Table 1:** miRNAs that promote HNSCC metastasis.

miRNA	Function/Target	Application	Reference
miRNA-134	WWOX	miRNA-134 expression enhances metastasis in mouse models by downregulating WWOX (tumor suppressor)	[37]
miRNA-155-5p	SOCS1, STAT3	miRNA-155-5p may induce EMT by upregulating STAT3 via SOCS1 downregulation	[38]
miRNA-196b	SOCS2	miRNA-196b promote invasion, suppress apoptosis by inhibiting SOCS2	[39]
miRNA-365a-3p	p-AKT	miRNA-365a-3p mediates the PI3K pathway by upregulating p-AKT to promote metastasis	[19]
miRNA-774-3p	PDCD4, PTEN, MMP-9	miRNA-744-3p suppresses PTEN, PDCD4 expression, enhancing MMP-9 and promoting EMT	[40]
miRNA-1246	DENND2D	miRNA-1246 regulates DENND2D, and upon suppression, DENND2D, promotes migration of human OSCC	[41]

**Table 2:** miRNAs that suppress HNSCC metastasis.

miRNA	Function/Target	Application	Reference
miRNA-29s (a/b/c)	LAMC2, ITGA6	miRNA-29s directly silences LAMC2 and ITGA6, inhibiting cell migration	[16]
miRNA-34a	NA	miRNA-34a transfection reduces invasion and CSC formation, downregulation of EMT	[22]
miRNA-101	EZH2	Suppression of miRNA-101 activates EZH2, inducing EMT and migration	[27]
miRNA-145-5p	FSCN1	miRNA-145-5p inhibited EMT and negatively regulates FSCN1, which promotes EMT in LSCC	[42]
miRNA-199 a/b	ITGA3	miRNA-199 family represses ITGA3 expression, leading to decreased migratory and invasion abilities	[14]
miRNA-200 a, b, 429	EZH2, STAT3	Overexpression of STAT3 globally silenced miRNA-200a, b, 429, but EZH2-depleted cells exhibited significantly increased miRNA-200-b/a/429 expression, which hindered EMT.	[43]
miRNA-203	PDPN	miRNA-203 directly inhibits PDPN, suppressing migration and LNM	[44]
miRNA-203	NUAK1	miRNA-203 suppresses the expression of NUAK1, which decreases invasion and LNM	[24]
miRNA-218-5p	CD44-ROCK	Downregulation of miRNA-218 increases invasion by activating CD44-ROCK	[45]
miRNA-26a/b, miRNA-29a/b/c, miRNA-218	LOXL2	Tumor suppressive miRNAs silence LOXL2 expression, which inhibits migration/invasion	[20]
miRNA-300	ET-1	miRNA-300 reduces ET-1 induced cell invasion	[46]
miRNA-363	PDPN	miRNA-363 downregulates PDPN to inhibit cell migration and invasion	[47]
miRNA-376c-3p	RUNX2, INHBA	Downregulation of miRNA-376c leads to the dysregulation of RUNX2/INHBA, which promotes metastatic progression	[18]
miRNA-876-5p	Vimentin	miRNA-876 downregulates vimentin, inhibiting metastasis	[48]

## Abbreviations

CSCs	Cancer stem cells
ECM	Extracellular matrix
EMT	Epithelial-mesenchymal transition
FSCN1	Fascin actin-bundling protein 1
HNSCC	Head and neck squamous cell carcinoma
HPSCC	Hypopharyngeal squamous cell carcinoma
HPV	Human papilloma virus
LNM	Lymph node metastasis
LSCC	Laryngeal squamous cell carcinoma
miRNA	Microrna
OPSCC	Oropharyngeal squamous cell carcinoma
OSCC	Oral squamous cell carcinoma
WWOX	WW domain-containing oxidoreductase
